# Anthropometric indices as predictors of hypertension among men and women aged 40–69 years in the Korean population: the Korean Genome and Epidemiology Study

**DOI:** 10.1186/s12889-015-1471-5

**Published:** 2015-02-13

**Authors:** Joung-Won Lee, Nam-Kyoo Lim, Tae-Hwa Baek, Sung-Hee Park, Hyun-Young Park

**Affiliations:** Division of Cardiovascular and Rare Diseases, Center for Biomedical Sciences, Korea National Institute of Health, 187 Osongsaengmyeng 2-ro, Osong-eup, Heungdeok-gu, Cheongju-si, Chungcheongbuk-do 361-951 Korea

**Keywords:** Obesity, Hypertension, Risk factors, Anthropometric indices

## Abstract

**Background:**

Obesity is one of the most significant risk factors for hypertension. However, there is controversy regarding which measure is the best predictor of hypertension risk. We compared body mass index (BMI), waist circumference (WC), waist-to-hip ratio (WHR), and waist-to-height ratio (WHtR) in subjects as predictive indicators for development of hypertension.

**Methods:**

The data were obtained from the Korean Genome and Epidemiology Study (KoGES), a large population-based prospective cohort study. A total of 4,454 subjects (2,128 men and 2,326 women) aged 40–69 years who did not have hypertension at baseline were included in this study. Incident hypertension was defined as systolic blood pressure ≥140 mmHg, diastolic blood pressure ≥90 mmHg, or anti-hypertensive medication use during the 4-year follow up. Receiver operating characteristic (ROC) analysis was used to compare discrimination abilities for anthropometric indices for hypertension. Hazard ratios were calculated by Cox proportional hazard model with adjustment for age, smoking status, alcohol consumption, diabetes and family history of hypertension by sexes.

**Results:**

In men, the area under the ROC curve (AROC) was 0.62 for WC, WHR, and WHtR and 0.58 for BMI. In women, the AROCs for BMI, WC, WHR, and WHtR were 0.57, 0.66, 0.68, and 0.68, respectively. After adjustment for risk factors, a 1 standard deviation increase in BMI, WC, WHR, WHtR were significantly related to incident hypertension, respectively (hazard ratio: 1.39, 1.50, 1.40 and 1.49 in men, 1.31, 1.44, 1.35 and 1.48 in women).

**Conclusions:**

The central obesity indices WC, WHR, and WHtR were better than BMI for the prediction of hypertension in middle-aged Korean people. WHtR facilitates prediction of incident hypertension because of the single standard regardless of sex, ethnicity, and age group. Therefore, WHtR is recommended as a screening tool for the prediction of hypertension.

## Background

Obesity increases the risk of hypertension, and the prevalence of obesity in middle-aged and elderly people has increased continuously [1-4]. In Korea, the prevalences of obesity among adults (age ≥30) as defined by body mass index (BMI) and waist circumference (WC) were reported to be 35.3% and 26.3%, respectively [4]. Notably, obesity can be defined by anthropometric indices, such as BMI, WC, waist-to-hip ratio (WHR), and waist-to-height ratio (WHtR). These anthropometric indices have been frequently used in epidemiological studies as they can be determined easily and at low cost [5]. BMI is the most widely used indicator of obesity, but it does not reflect central fat distribution, whereas WC, WHR, and WHtR are used as surrogate markers for body fat centralization [5-7]. A central distribution of body fat has been shown to be strongly associated with hypertension [5,8,9]. However, controversy remains regarding the best predictor of hypertension. Obesity has been defined as a BMI ≥30 kg/m^2^ in Western populations and ≥25 kg/m^2^ in Korean and other Asian populations [10]. Asians have higher body fat levels than Western people for the same BMI and WC according to epidemiologic studies on Asian populations [11,12]. The cut-off points for abdominal obesity in Korean adults were proposed to be a WC ≥90 cm in men and ≥85 cm in women by the Korean Society for the Study of Obesity (KSSO) [13]. The cut-off points of BMI for hypertension in men vary from 22.0 kg/m^2^ to 25.4 kg/m^2^ in different Asian countries [14]. This last study suggests the importance of applying ethnically appropriate cut-off points for anthropometric indices for hypertension. Most published research on obesity indices in relation to blood pressure is based on cross-sectional studies [2,8,13-17]. Only a few prospective studies of cut-off points for anthropometric indices for cardiovascular disease (CVD) have been conducted in Korea [18,19]. The purpose of this study was to evaluate and compare the abilities of BMI, WC, WHR, and WHtR as anthropometric indices to predict incident hypertension and to assess their associations in a Korean population aged 40 to 69 years.

## Methods

### Study population

**Figure 1:**
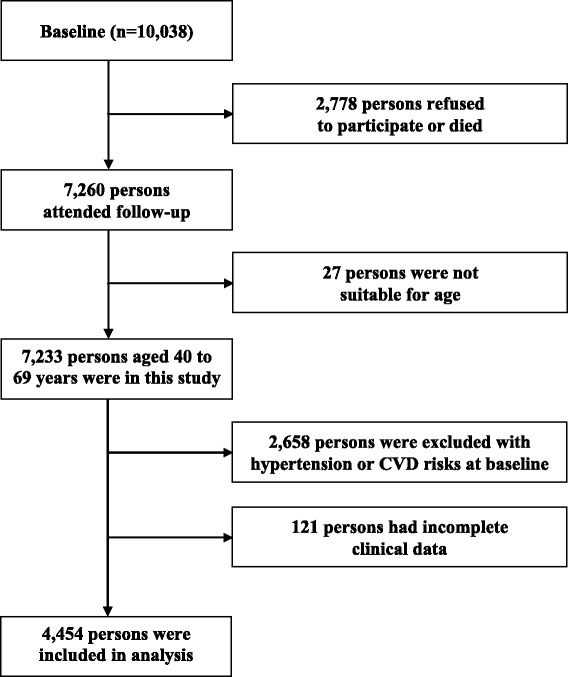
**Flow chart of study participants.**

The Korean Genome and Epidemiology Study (KoGES) is an ongoing community-based prospective cohort study of 10,038 participants. It was started in 2001 with the support of the Korean National Institute of Health. A baseline examination was performed on randomly selected participants in 2001–2002 and biennial follow-up examinations were subsequently conducted. The initial 2- and 4-year follow-up data are available to researchers (http://biomi.cdc.go.kr), and we obtained data for all participants from the Centers for Genome Science at the National Institute of Health, Korea. At the 2- and 4-year follow-up examinations, 7,260 participants were eligible in the present study after exclusion of 2,778 subjects who refused to participate or who had died. Of those participants, 7,233 aged 40 to 69 years were selected to participate in a baseline survey. We excluded 2,224 participants with hypertension at baseline. Additionally, 434 participants with previous CVD were excluded. Finally, after those with incomplete data were excluded, 4,454 participants remained eligible for this analysis (Figure 1). The criteria for exclusion based on hypertension at baseline were systolic blood pressure (SBP) ≥140 mmHg, diastolic blood pressure (DBP) ≥90 mmHg, or anti-hypertensive medication use. The study protocol was approved by the Institutional Review Board of the Korean Centers for Disease Control and Prevention.

### Measurements and surveys

Height and weight were measured (to the nearest 0.1 cm and 0.1 kg, respectively) using a digital stadiometer and scale. BMI (kg/m^2^) was calculated by dividing weight by height squared. WC was measured three times at the midpoint between the bottom of the ribcage and the top of the iliac crest using a fiberglass tape measure. Hip circumference was measured three times at the point of maximal protrusion of the buttocks; the mean of the three readings was considered the final hip circumference. WHR was calculated as WC divided by hip circumference and WHtR as WC divided by height. Blood pressure was measured in the sitting position after 5 min of rest using a standard mercury sphygmomanometer. Blood samples were obtained after fasting for at least 8 h. Fasting blood glucose, total cholesterol (TC), triglyceride (TG), and high-density lipoprotein cholesterol (HDL-C) levels were measured in a central, certified laboratory (Seoul Clinical Laboratories, Seoul, Republic of Korea). For subjects with TG levels <400 mg/dl, low-density lipoprotein cholesterol (LDL-C) levels were estimated indirectly using the Friedewald formula [20]. The questionnaire included questions on socio-demographic information, lifestyle, personal and family medical history, smoking status, and alcohol consumption. Smoking and alcohol consumption were defined as current smoker and current drinker, respectively.

### Definition of hypertension and diabetes mellitus

In the present study, patients with an SBP of ≥140 mmHg or a DPB of ≥90 mmHg, or who used anti-hypertensive medications, were defined as having hypertension. Diabetes mellitus was defined as a fasting blood glucose level of ≥126 mg/dl, a 2-h post-challenge plasma glucose (2 h-PCPG) level of ≥200 mg/dl, an HbA1c level of ≥6.5%, or use of oral hypoglycemic agents [21].

### Statistical analysis

Statistical analyses were performed using the SAS software (version 9.2; SAS Institute, Cary, North Carolina) and MedCalc (MedCalc Software, Mariakerke, Belgium). Continuous variables are expressed as the means ± SD, and discrete variables are expressed as counts and proportions. For comparisons between groups, Student’s *t*-test was used for continuous data and the chi-square test for categorical data. Receiver operating characteristic (ROC) analysis was used to compare discrimination abilities and to determine optimal cut-off values by sexes. Sensitivity (true-positive rate) and specificity (false-negative rate) based on cut-off values for the various anthropometric measurements and the overall discriminatory power of the diagnostic test were calculated using ROC curves. The cut-off points for hypertension were estimated using the maximized Youden index by sexes. The AUC of each obesity marker was compared those of BMI using the DeLong method [22]. We calculated hazard ratios (HRs) by Cox proportional hazard model with adjustment for age, smoking status, alcohol consumption, diabetes and family history of hypertension by sexes. The adjusted HRs are presented with 95% confidence intervals (CIs). The measurement units are different among obesity measures in the present study, so we compared the HRs according to a 1 standard deviation increase in each obesity parameter and criteria for obesity [10,13,23] by sexes. *P* < 0.05 was considered to indicate statistical significance.

## Results

### Baseline characteristics of the study subjects

The baseline characteristics of the study population, stratified by sex, are shown in Table 1. The mean age of the study population was 50.33 years in men and 50.21 years in women. The mean BMI was 23.93 kg/m^2^ in men and 24.36 kg/m^2^ in women. The mean WC was 82.45 cm in men and 79.88 cm in women. The mean WHR and WHtR were 0.88 and 0.49, respectively, in men and 0.86 and 0.52, respectively, in women. The prevalence of diabetes mellitus was higher in men than in women (6.67% *vs*. 5.16%, *P* < 0.032). Approximately 71.01% of the men were drinkers and about 50.19% were current smokers. 28.50% of women were drinkers and only 3.31% were current smokers.

**Table 1:** **Characteristics of the study population**

**Variables**	**Men (n = 2,128)**	**Women (n = 2,326)**	**P value**
Age (years)	50.33 ± 8.38	50.21 ± 8.30	0.6306
BMI (kg/m^2^)	23.93 ± 2.86	24.36 ± 3.09	<.0001
WC (cm)	82.45 ± 7.33	79.88 ± 9.12	<.0001
WHR	0.88 ± 0.06	0.86 ± 0.08	<.0001
WHtR	0.49 ± 0.04	0.52 ± 0.06	<.0001
SBP (mmHg)	113.80 ± 10.61	111.60 ± 11.90	<.0001
DBP (mmHg)	76.77 ± 7.39	73.73 ± 8.13	<.0001
FBG (mg/dl)	86.30 ± 15.40	82.20 ± 13.68	<.0001
TC (mg/dl)	190.40 ± 34.09	186.30 ± 33.21	<.0001
LDL-C (mg/dl)	115.20 ± 31.86	113.70 ± 29.26	0.1050
HDL-C (mg/dl)	43.66 ± 9.87	46.34 ± 9.66	<.0001
TG (mg/dl)	166.30 ± 106.20	133.10 ± 72.34	<.0001
Diabetes mellitus	142 (6.67)	120 (5.16)	0.0320
Drinker	1,511 (71.01)	663 (28.50)	<.0001
Smoker	1,068 (50.19)	77 (3.31)	<.0001

BMI, body mass index; WC, waist circumference; WHR, waist-to-hip ratio; WHtR. Waist-to-height ratio.

SBP, systolic blood pressure; DBP, diastolic blood pressure; FBG, fasting blood glucose; TC, total cholesterol.

LDL-C, low density lipoprotein cholesterol; HDL-C, high-density lipoprotein cholesterol; TG, triglycerides.

Values are mean ± standard deviation or n (%).

*P* values are from *t*-tests or chi-square tests for analysis of variance for continuous variables and categorical variables.

### Incidence of hypertension according to anthropometric index categories

During the 4-year follow up, the overall cumulative incidence of hypertension was 18.05% (384 cases, 50.20 cases/1,000 person-years) in men and 16.08% (374 cases, 43.87 cases/1,000 person-years) in women. As shown in Table 2, the number of cases and incidence rate of hypertension significantly increased with increasing anthropometric index values in both sexes.

**Table 2:** **Number of incident hypertension, person-years of follow-up by category of each anthropometric index**

**Anthropometric indices**	**Men (n = 2,128)**					**Women (n = 2,326)**				
	**Quartile**	**N**	**Event**	**Person-years**	**Incidence rate/1,000 person-years**	**Quartile**	**N**	**Event**	**Person-years**	**Incidence rate/1,000 person-years**
BMI(kg/m^2^)	Q1 (<21.97)	532	70	1,956.37	35.78	Q1 (<22.28)	581	77	2,152.03	35.78
	Q2 (21.97-23.93)	532	83	1,923.07	43.16	Q2 (22.28-24.16)	582	77	2,173.86	35.42
	Q3 (23.94-25.84)	532	107	1,906.85	56.11	Q3 (24.17-26.16)	581	92	2,129.02	43.21
	Q4 (≥25.85)	532	124	1,863.28	66.55	Q4 (≥26.17)	582	128	2,070.47	61.82
WC(cm)	Q1 (<77.33)	528	61	1,941.20	31.42	Q1 (<73.00)	547	42	2,065.16	20.34
	Q2 (77.33-82.49)	533	79	1,950.32	40.51	Q2 (73.00-78.99)	593	63	2,233.75	28.20
	Q3 (82.50-87.66)	554	112	1,975.22	56.70	Q3 (79.00-86.16)	607	112	2,198.71	50.94
	Q4 (≥87.67)	513	132	1,782.83	74.04	Q4 (≥86.17)	579	157	2,027.75	77.43
WHR	Q1 (<0.85)	534	56	1,968.62	28.45	Q1 (<0.79)	582	30	2,224.95	13.48
	Q2 (0.85-0.87)	531	84	1,915.97	43.84	Q2 (0.79-0.84)	581	68	2,168.85	31.35
	Q3 (0.88-0.91)	531	101	1,905.00	53.02	Q3 (0.85-0.91)	582	123	2,083.68	59.03
	Q4 (≥0.92)	532	143	1,859.99	76.88	Q4 (≥0.92)	581	153	2,047.89	74.71
WHtR	Q1 (<0.46)	532	58	1,961.34	29.57	Q1 (<0.47)	582	36	2,212.78	16.27
	Q2 (0.46-0.48)	532	78	1,935.73	40.29	Q2 (0.47-0.50)	581	67	2,169.60	30.88
	Q3 (0.49-0.52)	532	109	1,907.64	57.14	Q3 (0.51-0.55)	581	109	2,109.73	51.67
	Q4 (≥0.52)	532	139	1,844.87	75.34	Q4 (≥0.56)	582	162	2,033.27	79.67
Total		2,128	384	7,649.58	50.20		2,326	374	8,525.38	43.87

BMI, body mass index; WC, waist circumference; WHR, waist-to-hip ratio; WHtR, waist-to-height-ratio.

### Cut-off points for the various anthropometric indices for predicting hypertension

Table 3 shows the AROC values and cut-off points for the anthropometric indices for predicting hypertension. In men, the AROC for WC, WHR, and WHtR was 0.62 while the AROC for BMI was 0.58. In women, the AROCs for BMI, WC, WHR, and WHtR were 0.57, 0.66, 0.68, and 0.68, respectively. In both sexes, the AROC for BMI was smaller than the AROC for the central obesity indices. The AROCs for WC, WHR, and WHtR were significantly different (*P* < 0.01) compared to the AROC for BMI in both sexes. The optimal cut-off points for predicting hypertension using the Youden index were 23.59 kg/m^2^, 83.33 cm, 0.88, and 0.49 in men and 25.63 kg/m^2^, 80.37 cm, 0.86, and 0.51, in women for BMI, WC, WHR, and WHtR, respectively.

**Table 3:** **The area under the ROC curve (AROC) and cut-off points for anthropometric indices to predict the hypertension**

**Anthropometric index**	**AROC (95% CI)**	**Cut-off point**	**Sensitivity (%)**	**Specificity (%)**	**Youden index (95% CI)** ^**a)**^
***Men***					
BMI (kg/m^2^)	0.58 (0.56 ~ 0.60)	23.59	66.15	47.42	0.14 (0.10 ~ 0.20)
WC (cm)	0.62 (0.60 ~ 0.64)***	83.33	59.90	57.91	0.18 (0.14 ~ 0.24)
WHR	0.62 (0.60 ~ 0.64)**	0.88	67.97	49.66	0.18 (0.14 ~ 0.24)
WHtR	0.62 (0.60 ~ 0.64)***	0.49	69.27	48.91	0.18 (0.15 ~ 0.24)
***Women***					
BMI (kg/m^2^)	0.57 (0.55 ~ 0.59)	25.63	42.78	71.21	0.14 (0.10 ~ 0.20)
WC (cm)	0.66 (0.64 ~ 0.68)***	80.37	66.04	60.45	0.26 (0.22 ~ 0.32)
WHR	0.68 (0.66 ~ 0.70)***	0.86	71.12	57.94	0.29 (0.25 ~ 0.35)
WHtR	0.68 (0.66 ~ 0.70)***	0.51	75.13	53.18	0.28 (0.24 ~ 0.33)

CI, confidence interval; BMI, body mass index; WC, waist circumference; WHR, waist-to-hip ratio; WHtR, waist-to-height-ratio.

^a)^95% confidence intervals of Youden index were based on 10,000 bootstrap samples.

**P <0.01, ***P <0.001 *vs* BMI.

### HRs for hypertension according to anthropometric index

Table 4 shows that the trends were similar among the HRs for each 1-unit increase in standard deviation for each obesity parameter.

**Table 4:** **Association between various anthropometric indices and incident hypertension**

**Anthropometric indices**	**Model 1**	**Model 2**	**Model 3**
	**HR (95% CI)**	**HR (95% CI)**	**HR (95% CI)**
***Men***			
BMI (kg/m^2^)	1.28 (1.16 ~ 1.42)	1.38 (1.24 ~ 1.53)	1.39 (1.25 ~ 1.54)
WC (cm)	1.47 (1.33 ~ 1.63)	1.50 (1.36 ~ 1.66)	1.50 (1.36 ~ 1.67)
WHR	1.44 (1.31 ~ 1.59)	1.40 (1.27 ~ 1.55)	1.40 (1.27 ~ 1.55)
WHtR	1.49 (1.35 ~ 1.65)	1.48 (1.34 ~ 1.63)	1.49 (1.35 ~ 1.65)
***Women***			
BMI (kg/m^2^)	1.27 (1.15 ~ 1.40)	1.30 (1.18 ~ 1.43)	1.31 (1.19 ~ 1.44)
WC (cm)	1.66 (1.51 ~ 1.83)	1.43 (1.29 ~ 1.58)	1.44 (1.30 ~ 1.60)
WHR	1.67 (1.52 ~ 1.83)	1.35 (1.21 ~ 1.50)	1.35 (1.21 ~ 1.51)
WHtR	1.76 (1.60 ~ 1.94)	1.46 (1.32 ~ 1.62)	1.48 (1.33 ~ 1.64)

HR, hazard ratio; CI, confidence interval; BMI, body mass index; WC, waist circumference; WHR, waist-to-hip ratio; WHtR, waist-to-height-ratio.

Model 1, unadjusted. Model 2, adjusted for age. Model 3, adjusted for age, smoking status, alcohol consumption, diabetes and family history of hypertension.

HR per 1 standard deviation (2.86 kg/m^2^ in men and 3.09 kg/m^2^ in women, 7.33 cm in men and 9.12 cm in women, 0.06 in men and 0.08 in women, 0.04 in men and 0.06 in women) increment of BMI, WC, WHR or WHtR.

The HR for BMI was lower than those for the central obesity markers WC, WHR, and WHtR in their associations with incident hypertension in both sexes.

After adjustment for age, smoking status, alcohol consumption, diabetes and family history of hypertension, anthropometric indices showed no significant associations, but an increasing trend similar to that for the unadjusted model was maintained. Table 5 shows the HRs for incident hypertension according to obesity status defined by anthropometric indices.

**Table 5:** **Association between various anthropometric indices and incident hypertension according to the obesity status**

**Variables**	**Men (n = 2,128)**			**Women (n = 2,326)**		
	**Model 1**	**Model 2**	**Model 3**	**Model 1**	**Model 2**	**Model 3**
	**HR (95% CI)**	**HR (95% CI)**	**HR (95% CI)**	**HR (95% CI)**	**HR (95% CI)**	**HR (95% CI)**
BMI ≥ 25 kg/m^2a)^	1.54 (1.26 ~ 1.88)	1.69 (1.38 ~ 2.08)	1.67 (1.35 ~ 2.05)	1.56 (1.27 ~ 1.90)	1.54 (1.26 ~ 1.89)	1.54 (1.26 ~ 1.89)
WC ≥ 90/85 cm^b)^	2.12 (1.69 ~ 2.66)	2.15 (1.71 ~ 2.70)	2.14 (1.70 ~ 2.69)	2.29 (1.87 ~ 2.81)	1.68 (1.36 ~ 2.07)	1.69 (1.37 ~ 2.08)
WHR ≥ 0.9/0.85^c)^	1.71 (1.40 ~ 2.09)	1.63 (1.33 ~ 1.99)	1.60 (1.31 ~ 1.96)	3.24 (2.56 ~ 4.10)	2.13 (1.66 ~ 2.75)	2.13 (1.65 ~ 2.74)
WHtR ≥ 0.5	1.89 (1.55 ~ 2.32)	1.87 (1.52 ~ 2.29)	1.86 (1.51 ~ 2.28)	3.30 (2.55 ~ 4.26)	2.24 (1.71 ~ 2.93)	2.22 (1.70 ~ 2.90)

^a)^BMI, WHO (2000).

^b)^WC ≥ 90 cm for men and ≥ 85 cm for women, Korean Society for the Study of Obesity (2007).

^c)^WHR ≥ 0.9 for men and ≥ 0.85 for women, WHO (1999).

HR, hazard ratio; CI, confidence interval; BMI, body mass index; WC, waist circumference; WHR, waist-to-hip ratio.

WHtR, waist-to-height-ratio.

Model 1, unadjusted. Model 2, adjusted for age. Model 3, adjusted for age, smoking status, alcohol consumption, diabetes and family history of hypertension.

After adjustment for risk factors, the hazard ratios for BMI, WC, WHR and WHtR were 1.39, 1.50, 1.40 and 1.49 in men, 1.31, 1.44, 1.35 and 1.48 in women, respectively. In both sexes, the HRs for hypertension according to BMI were lower than those for the anthropometric indices related to central obesity.

## Discussion

We analyzed the usefulness of anthropometric indices as predictors of hypertension. In the present study, the AROC for BMI was smaller than that for WC, WHR, and WHtR, suggesting that anthropometric indices that reflect central obesity are better for predicting hypertension in both sexes. Our results are in accordance with previous comparative studies of the association between obesity measures and hypertension [8,24,25]. The HRs for BMI were also lower than those for central obesity indices, with and without adjustment, in both sexes, similar to the AROC results. The discrimination ability of WHtR was similar to that of WC and WHR in our study, however WHtR is more convenient than other anthropometric indices. The sex differences in cut-off points were smaller for WHR and WHtR than for BMI and WC. The result of WHtR was consistent with our previous study which was cross-sectional using data of the Third Korea National Health and Nutrition Examination Survey (KNHANES III) [25]. Unfortunately however, WHR was not included in our previous study. The small sex difference is based on the same standard, which is easy to memorize and consumer-friendly. Ashwell et al. suggest a public message for adults to prevent hypertension: “keep your WC below your half height” [26]. Tseng et al. assert that these characteristics of WHtR also apply to different ethnic groups, which make it convenient for international research [27]. Moreover, WHtR is useful for children, which makes it suitable for long-term follow-up over the lifetime of an individual. Ashwell et al. investigated the relationship between CVD and anthropometric indices by meta-analysis. Discrimination of hypertension using WHtR was 3–4% better than with BMI [28]. On the other hand, WC and WHR have disadvantages. These anthropometric indices do not reflect the height of the subject [7,8]. Hsieh et al. reported that in Japanese men in the third quartile of WC (84.5– < 89 cm) short individuals had a greater risk of hypertension than those who were taller [29]. Moreover, measuring hip circumference is more difficult than measuring WC, and accurately identifying the point of maximal protrusion of the buttocks in obese people is demanding [27,30]. Previous studies reported that the AROC for WHR for hypertension was the lowest among the anthropometric indices [16,27]. Above all, WHR and WC are not consumer-friendly because of the different cut-off points according to sex [8,26,27]. Considering our results and previous studies, WHtR is an affordable screening tool for predicting hypertension in Korean adults. The present study had a number of strengths. Firstly, it used a large population-based sample and a prospective cohort design. Therefore, the causality between anthropometric indices and incident hypertension is clear. Secondly, interviews were conducted by trained interviewers and anthropometric data were obtained by repeated measurement using a standard protocol. These processes may have helped to reduce bias. The fact that dietary intakes and physical activity were not considered in the analysis is a limitation of the present study. These variables were reported to be risk factors for incident hypertension in previous studies [31,32]. Another limitation in present study is that blood pressure is measured during the visit of each follow-up. Repeated blood pressure measurements during two or more visits are recommended by the Seventh Report of the Joint National Committee on the Prevention, Detection, Evaluation, and Treatment of High Blood Pressure (JNC 7) [33].

## Conclusions

In conclusion, WHtR can be recommended as a useful screening tool for predicting hypertension because of its high discrimination ability. Moreover, the cut-off points for WHtR for hypertension were similar in both sexes. WHtR is user-friendly and can be converted into a public message.

## Abbreviations

BMI: Body mass index
WC: Waist circumference
WHR: Waist-to-hip ratio
WHtR: Waist-to-height ratio
KoGES: Korean Genome and Epidemiology Study
ROC: Receiver operating characteristic
AROC: Area under the ROC curve
KSSO: Korean Society for the Study of Obesity
CVD: Cardiovascular disease
SBP: Systolic blood pressure
DBP: Diastolic blood pressure
TC: Total cholesterol
TG: Triglyceride
HDL-C: High-density lipoprotein cholesterol
LDL-C: Low-density lipoprotein cholesterol
2 h-PCPG: 2-h post-challenge plasma glucose
HRs: Hazard ratios
CIs: Confidence intervals
KNHANES: Korea National Health and Nutrition Examination
